# Interobserver agreement of the Taulí-pT1 classification in rectal pT1 adenocarcinoma

**DOI:** 10.1007/s00384-025-04996-6

**Published:** 2025-09-17

**Authors:** Cristina Gener-Jorge, Joan Carles Ferreres Piñas, Ana Belén Moreno Garcia, Doris Sofia Melgar Rivera, Alex Casalots, Anna Nonell, Beatriz Espina, Aleidis Caro-Tarragó, Xavier Serra-Aracil

**Affiliations:** 1 Department of Pathology, Institut d’Investigació I Innovació Parc Taulí (I3PT-CERCA), Parc Taulí Hospital Universitari, Universitat Autònoma de Barcelona, Sabadell, Spain; 2 Department of Pathology, Consorci del Laboratori Intercomarcal de L’Alt Penedés I El Garraf, CLILAB Diagnostics, Barcelona, Spain; 3 Department of Surgery, Parc Taulí Hospital Universitari, Institut d’Investigació I Innovació Parc Taulí (I3PT-CERCA), Universitat Autònoma de Barcelona, Sabadell, Spain; 4 Department of Surgery, Hospital de La Santa Creu I Sant Pau, Institut de Recerca Sant Pau (IR Sant Pau), Universitat Autònoma de Barcelona, Barcelona, Spain; 5 Department of Surgery, Hospital Universitari Joan XXIII, Tarragona, Spain; 6 Department of Surgery, Universitat Autònoma de Barcelona, Barcelona, Spain

**Keywords:** T1 rectal adenocarcinoma, Submucosal invasion, Prognosis of T1 rectal adenocarcinoma, Healthy residual submucosa, TEM

## Abstract

**Purpose:**

In pT1 rectal adenocarcinoma, adverse pathological features guide the indication for radical surgery; however, they are present in only 10–15% of cases. Therefore, in most patients, accurate en bloc local excision with clear margins and precise submucosal invasion assessment is essential for appropriate risk stratification. Beyond absolute depth, a major challenge is the wide interindividual variability in submucosal thickness. The Taulí-pT1 classification, based on the measurement of healthy residual submucosa (hrSB) from the muscularis propria, has been proposed as an objective and reproducible system. This study aimed to validate this classification.

**Method:**

An interobserver study was conducted on 30 patients with pT1 rectal adenocarcinoma treated by transanal endoscopic surgery. Four pathologists with varying experience levels independently evaluated digitized histological slides, measuring hrSB, total submucosal thickness, and invasion depth. They also classified specimens as sm1, sm2, or sm3. Interobserver agreement was assessed using intraclass correlation coefficients (ICC), and Fleiss’ and Cohen’s kappa indices.

**Results:**

Excellent interobserver agreement was observed for hrSB (ICC = 0.99; 95% CI: 0.98–0.99), total submucosal thickness (ICC = 0.96; 95% CI: 0.93–0.98), and depth of invasion (ICC = 0.94; 95% CI: 0.9–0.97). The Taulí-pT1 classification demonstrated good agreement (Fleiss’ kappa = 0.71). Identification of the muscularis mucosae showed moderate agreement (kappa = 0.612).

**Conclusion:**

The Taulí-pT1 classification demonstrates high interobserver reproducibility, even among pathologists with varying levels of experience, supporting its utility as an objective and standardized tool for the assessment of pT1 rectal adenocarcinoma.

**Clinical trial registration:**

ClinicalTrials.gov Identifier: NCT06218108.

**Supplementary Information:**

The online version contains supplementary material available at 10.1007/s00384-025-04996-6.

## Introduction

The early diagnosis of colorectal cancer has significantly increased over recent decades, driven by the implementation of population-based screening programmes. As a result, there has been a rise in the detection of early-stage tumors, including pT1 rectal adenocarcinomas, which are characterized by invasion limited to the submucosa [1, 2].

The optimal management of these lesions relies on accurate risk stratification for lymph node involvement, which determines whether treatment should be conservative—via local excision—or more radical, through total mesorectal excision. Current clinical guidelines, both Japanese (Japanese Society for Cancer of the Colon and Rectum, JSCCR) [3] and European (European Society of Gastrointestinal Endoscopy, ESGE) [4], consider high-risk features to include submucosal invasion ≥ 1000 µm, lymphovascular invasion, poor differentiation, and tumor budding.

However, these adverse pathological features are present in fewer than 10–15% of pT1 [5, 6] rectal adenocarcinomas. Therefore, in most cases, a proper en bloc local excision and the depth of submucosal invasion remain the primary criteria for guiding therapeutic decisions. Various methods—both qualitative and quantitative—have been proposed to measure this invasion. Nevertheless, their clinical applicability has been limited by issues related to reproducibility, morphological variability, and reliance on inconsistent anatomical landmarks such as the muscularis mucosae [2, 5].

Moreover, one of the main limitations of absolute invasion measurements is the assumption that submucosal thickness is uniform across patients, whereas in reality it shows considerable anatomical variability. This heterogeneity compromises the validity of fixed thresholds as universal prognostic criteria and may lead to inaccuracies in risk estimation.

To overcome these limitations, the Taulí-pT1 classification was proposed by Casalots et al. [5], based on measuring the healthy residual submucosa (hrSB) from the muscularis propria. This approach aims to provide a more objective and standardized system, independent of lesion morphology or the presence of variable histological structures.

The aim of the present study is to validate the Taulí-pT1 classification through an interobserver agreement analysis evaluating its reproducibility among pathologists with different levels of experience in order to assess its applicability in routine clinical practice.

## Materials and methods

### Study design

An analytical, retrospective observational study was conducted to assess interobserver agreement in the application of the Taulí-pT1 classification for pT1 rectal adenocarcinoma. The study followed the STROBE guidelines [7] for observational research, aiming to ensure a clear, comprehensive, and reproducible description of the methods employed.

### Setting and study period

The study was carried out at a tertiary university hospital and included 30 consecutive cases recorded in the institutional pathology database. All patients were treated with transanal endoscopic surgery, either transanal endoscopic microsurgery (TEM) or transanal endoscopic operation (TEO), and had a histopathological diagnosis of pT1 rectal adenocarcinoma.

### Participants and selection criteria

Inclusion criteria: Consecutive cases treated with local excision via transanal endoscopic surgery (TEM/TEO), with a confirmed diagnosis of pT1 rectal adenocarcinoma.

Exclusion criteria: Lesions located outside the rectum, histologies not compatible with adenocarcinoma, incomplete resections, or specimens lacking clear visualization of the muscularis propria.

### Measurement procedure

All slides from each case were reviewed, and the section showing tumor invasion closest to the muscularis propria was selected. This section was digitized to allow precise micrometric measurement. The four participating pathologists—one junior (Pat1-CR), two gastrointestinal pathology specialists (Pat2-AB, Pat3-SM), and one senior generalist (Pat4-JC)—performed the measurements independently and blinded to each other's results.

Prior to analysis, the observers reviewed and agreed upon the measurement protocol in order to standardize technical criteria and reduce variability derived from the measurement process (see video).

**Fig. 1:**
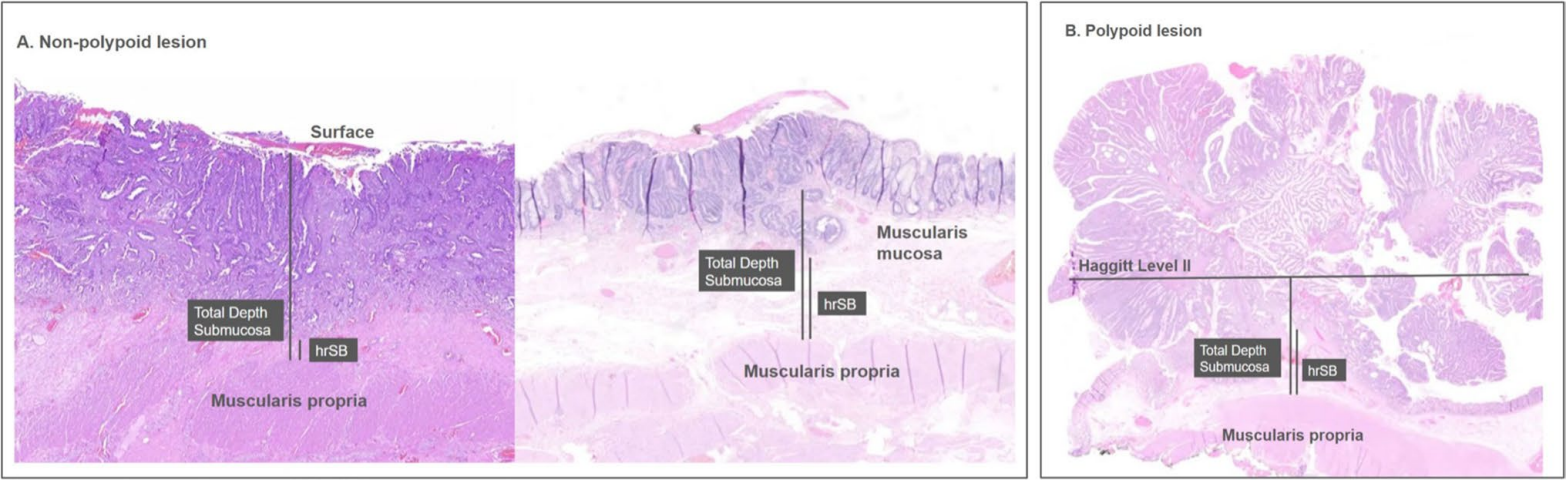
Measurement of submucosal invasion and healthy residual submucosa (hrSB). **A** Non-polypoid lesion: left image shows a sessile lesion without identifiable muscularis mucosae; right image includes muscularis mucosae. **B** Polypoid lesion: submucosal depth and hrSB are measured using Haggitt Level II as the surface reference

The healthy residual submucosa (hrSB) was defined as the distance between the muscularis propria and the deepest point of tumor invasion. The total submucosal thickness was measured from the muscularis propria to the surface (either to the muscularis mucosae, if present, or to the superficial edge of the lesion if absent) [5] (Fig. 1A). In polypoid lesions, the reference point was the neck of the polyp or Haggitt level II [8] (Fig. 1B). Using these measurements, the inverse percentage of invasion relative to the hrSB was calculated as follows: (total submucosal thickness – hrSB)/100. Based on this percentage, qualitative sm categories were assigned by each pathologist: sm1 (< 33.3%), sm2 (33.3%–66.6%), and sm3 (> 66.6%).

### Variables

The primary variable was tumor invasion depth (in µm), calculated using the healthy residual submucosa (hrSB) according to the Taulí-pT1 method [5].

Secondary variables included: quantitative variables: total submucosal thickness and depth of invasion. qualitative variables: presence or absence of the muscularis mucosae, and sm category (sm1, sm2, sm3), based on the Taulí-pT1 measurement method.

### Sample size and bias reduction measures

A sample size of 30 patients was selected based on methodological standards for interobserver agreement and validation studies, where this number is considered sufficient to assume normal distribution and the applicability of parametric tests—placing it within the range of large samples for this type of analysis [9].

The following measures were taken to minimize bias: Consecutive case selection to avoid selection bias; a standardized measurement protocol and prior training of the pathologists; blinding of observers to the measurements made by other participants.

### Statistical analysis

Statistical analysis was performed using SPSS (version 26).

Continuous variables were expressed as mean ± standard deviation or as median and interquartile range (IQR), depending on distribution normality. Categorical variables were reported as absolute and relative frequencies. Normality was assessed using the Kolmogorov–Smirnov and Shapiro–Wilk tests.

To evaluate interobserver agreement for continuous variables, Fisher’s intraclass correlation coefficient (ICC) was used. The ICC assesses the overall agreement between two or more measurement methods based on an analysis of variance (ANOVA) model for repeated measures. Agreement was interpreted as follows: < 0.5 = poor; 0.5–0.75 = moderate; 0.75–0.9 = good; > 0.9 = excellent.

Cohen’s kappa index was used for agreement between two observers, while Fleiss’ kappa index was applied for multiple observers and/or categories. Fleiss’ kappa was calculated using a customized Microsoft Excel® spreadsheet in which the classification assigned by each evaluator to each item was entered. Interpretation of kappa values was as follows: < 0.2 = slight; 0.21–0.4 = fair; 0.41–0.6 = moderate; 0.61–0.8 = substantial; > 0.8 = excellent.

Statistical significance was set at *p* < 0.05 with a 95% confidence interval.

### Ethical considerations

The study protocol was approved by the Clinical Research Ethics Committee (CEIm: 2023/5123) and complied with the principles of the Declaration of Helsinki for research involving human subjects.

## Results

A total of 30 patients with pT1 rectal adenocarcinoma treated by transanal endoscopic surgery were analyzed. The mean age was 71.4 years, with an equal sex distribution (15 men and 15 women). Regarding tumour morphology, most lesions were sessile (13 cases, 43.3%), and 8 (26.7%) presented a polypoid morphology. The median tumor size was 3.5 cm, and the mean distance from the anal verge was 8 cm. Histologically, 15 lesions (50%) were adenocarcinomas (ADKs) without adjacent adenomatous components, while 13 cases (43.3%) showed adenocarcinoma with associated high-grade dysplasia. Most tumours were well (G1) or moderately (G2) differentiated (28 of 30, 93.3%). The prevalence of adverse prognostic features was low: lymphatic invasion in 2 cases (6.7%), perineural invasion in 1 case (3.3%), and tumour budding in 1 case (3.3%) (Table 1).

**Table 1:** Demographic, preoperative, and pathological characteristics of patients

Variable			Results (*n* = 30)
Demographic	Age (mean ± SD), years		71.4 ± 1.1
	Sex, *n* (%)	Male	15 (50%)
		Female	15 (50%)
Tumor-related variables	Distance from anal verge (median, IQR) cm		8 (4.3)
	Tumor size (median, IQR) cm		3.5 (4.3)
	Size by number of quadrants, *n* (%)	1 quadrant	18 (60%)
		2 quadrants	11 (36.7%)
		3–4 quadrants	1 (3.3%)
	Lesion morphology, *n* (%)	Flat	7 (23.3%)
		Polypoid	8 (26.7%)
		Sessile	13 (43.3%)
		Ulcerated	2 (6.7%)
	Tumour location by quadrant, *n* (%)	Anterior	5 (16.7%)
		Left lateral	7 (23.3%)
		Right lateral	13 (43.3%)
		Posterior	5 (16.7%)
Pathological features	ADK subtype (with or without adenomatous component) *n* (%)	ADK with low-grade dysplasia	2 (6.7%)
		ADK with high-grade dysplasia	13 (43.3%)
		Pure ADK (no adenomatous component)	15 (50%)
	Degree of differentiation, *n* (%)	G1–G2	28 (93.3%)
		G3–G4	2 (6.7%)
	Adverse pathological features, *n* (%)	Lymphatic invasion	2 (6.7%)
		Perineural invasion	1 (3.3%)
		Vascular invasion	0 (0%)
		Tumor budding	1 (3.3%)

*SD* standard deviation, *IQR* interquartile range, *ADK* adenocarcinoma

**Fig. 2:**
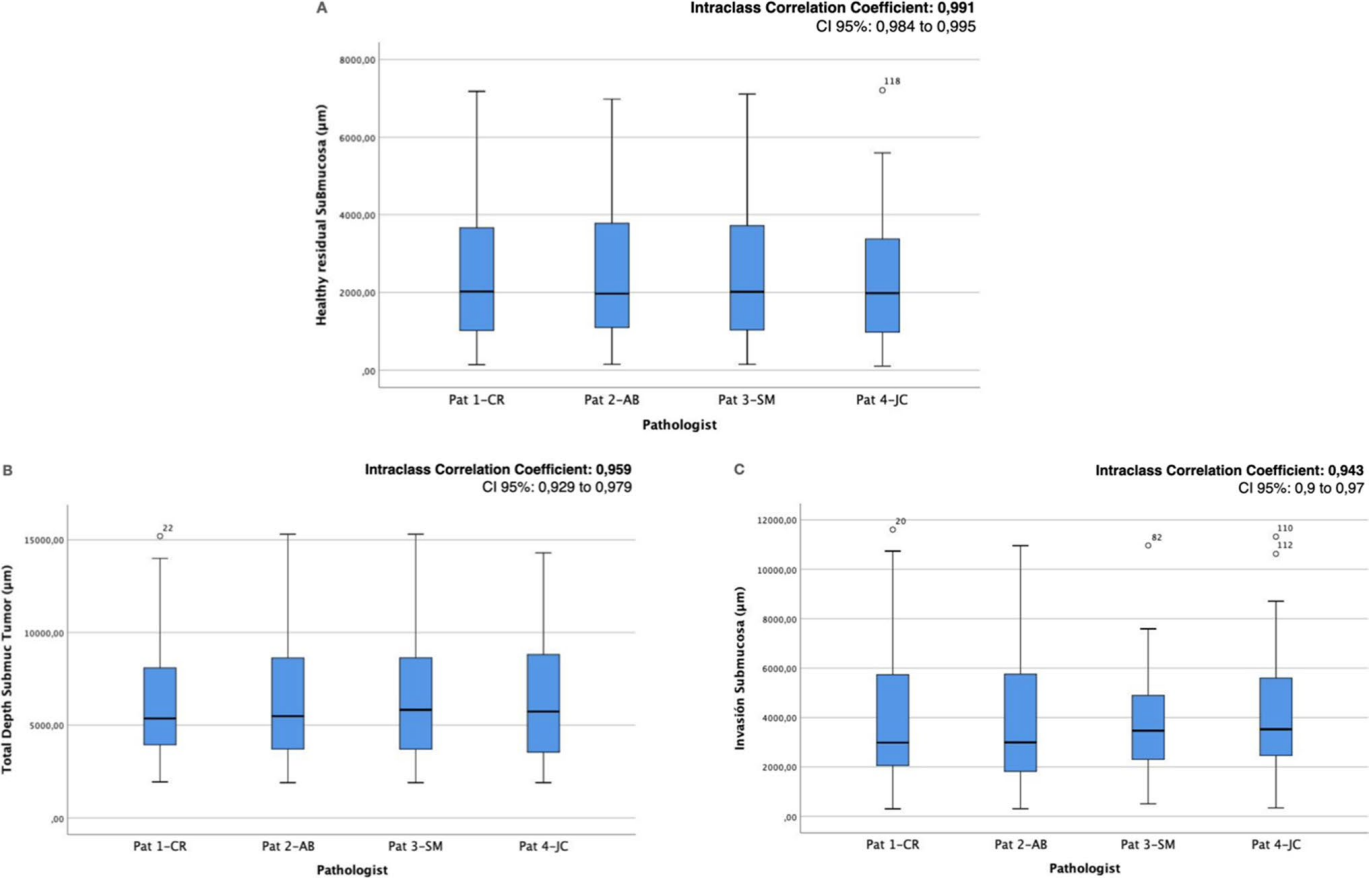
Boxplots showing intra-class correlation coefficients (ICC) among the four pathologists for the three main quantitative variables: **A** healthy residual submucosa (hrSB); **B** total submucosal thickness; **C** tumor submucosal invasion

The quantitative variables analyzed—healthy residual submucosa (hrSB), total submucosal thickness, and depth of invasion from the surface—mostly showed a normal distribution according to the Kolmogorov–Smirnov test. However, they were described using median, interquartile range (IQR), and full range to maintain consistency in result presentation, as shown in the corresponding figures (Fig. 2).

Regarding the primary variable, hrSB measurements showed excellent interobserver agreement among the four pathologists, with a global intraclass correlation coefficient (ICC) of 0.99 (95% CI 0.98–0.99) (Table 2). Pairwise correlations between observers ranged from 0.970 to 0.988, indicating outstanding measurement consistency (Fig. 2A). Submucosal invasion also demonstrated a high level of agreement, with a global ICC of 0.94 (95% CI 0.9–0.97). As for total submucosal thickness in the tumour area, a wide range of variability was observed among lesions, with values ranging from 1900 to 15,300 µm. Nevertheless, interobserver agreement for this measurement was also excellent, with a global ICC of 0.96 (95% CI 0.93–0.98). Figure 2 (boxplots) graphically represents the measurements recorded by each observer, showing similar central distributions and narrow interquartile ranges for all three quantitative variables.

**Table 2:** Interobserver intraclass correlation coefficient (ICC) for hrSB, total submucosal thickness, and submucosal invasion

Variable	Pat 1-CR	Pat 2-AB	Pat 3-SM	Pat 4-JC	ICC (95% CI)
A. Healthy residual submucosa (hrSB) (µm)					
hrSB (median [IQR], [range])	2021.6 (2699.2) [142.7–7180.6]	1968.3 (2729.6) [151–6979.9]	2013.9 (2820.7) [151–7111]	2030.7 (2598.3) [104.1–7207.5]	0.99 (0.98–0.99)
Pat 2-AB	0.99 (0.97–0.99)				
Pat 3-SM	0.98 (0.97–0.99)	0.98 (0.96–0.99)			
Pat 4-JC	0.986 (0.971–0.994)	0.98 (0.96–0.99)	0.970 (0.94–0.99)		
B. Total submucosal thickness (µm)					
Total thickness (median [IQR], [range])	5353.1 (4252.2) [1945–15,200]	5480.1 (5032.9) [1900–15,300]	5818.4 (5046.1) [1900–15,300]	5311.6 (5399.45) [1899–14,300]	0.96 (0.93–0.98)
Pat 2-AB	0.991 (0.98–0.99)				
Pat 3-SM	0.95 (0.89–0.98)	0.957 (0.91–0.980)			
Pat 4-JC	0.91 (0.79–0.95)	0.88 (0.74–0.94)	0.85 (0.683–0.928)		
C. Submucosal invasion (µm)					
Invasion (median [IQR], [range])	2983.1 (3756.7) [306–11,605.7]	2992.2 (4331.6) [309.5–10,957.1]	3468.1 (2805.55) [507.5–10,962]	3328.35 (3518.4) [342.3–11,319]	0.94 (0.9–0.97)
Pat 2-AB	0.986 (0.97–0.99)				
Pat 3-SM	0.894 (0.78–0.95)	0.91 (0.81–0.96)			
Pat 4-JC	0.88 (0.75–0.94)	0.87 (0.73–0.94)	0.78 (0.53–0.89)		

*CI* confidence interval, *IQR* interquartile range

Regarding the categorical variables, the presence of the muscularis mucosa was observed in a low percentage of cases (ranging from 13.3% to 23.3%, depending on the observer), limiting its usefulness on its own as an anatomical reference point for measuring submucosal invasion. Interobserver agreement for this variable was moderate, with a Fleiss’ kappa index of 0.61 (95% CI: 0.47–0.75) (Table 3). Cohen’s kappa values for pairwise comparisons among pathologists ranged from 0.31 to 0.81, indicating considerable heterogeneity in its identification, likely due to its inconsistent presence and the subjectivity involved in its histological interpretation.

**Table 3:** Interobserver agreement on muscularis mucosa identification and Sm1–Sm3 Taulí-pT1 classification

Muscularis mucosa (presence or absence)				
Fleiss’ Kappa Index (Pat1-CR, Pat2-AB, Pat3-SM, Pat4-JC) (95% CI)			0.61 (95% CI 0.466 to 0.75)	
Pathologist	Pat1-CR	Pat2-AB	Pat3-SM	Pat4-JC
Presence of muscularis mucosa (*n*/30, %)	7 (23.3%)	6 (20%)	4 (13.3%)	5 (16.7%)
Cohen’s Kappa Index between pairs of pathologists				
Pat1-CR	-	0.814	0.51	0.793
Pat2-AB	0.814	-	0.314	0.586
Pat3-SM	0.51	0.314	-	0.667
Pat4-JC	0.793	0.586	0.667	-
Sm1–Sm3 classification (Taulí-pT1)				
Fleiss’ Kappa Index (Pat1-CR, Pat2-AB, Pat3-SM, Pat4-JC) (95% CI)			0.71 (95% CI 0.6 to 0.82)	
Cohen’s Kappa Index between pairs of pathologists				
	P Pat1-CR	Pat2-AB	Pat3-SM	Pat4-JC
Pat1-CR	-	0.73	0.67	0.73
Pat2-AB	0.73	-	0.73	0.73
Pat3-SM	0.67	0.73	-	0.66
Pat4-JC	0.73	0.73	0.66	-

*CI* confidence interval

Classification of lesions according to the Taulí-pT1 scale (Sm1, Sm2, Sm3) showed substantial agreement, with a Fleiss’ kappa of 0.71 (95% CI 0.6–0.82). Pairwise kappa values among pathologists ranged from 0.68 to 0.73, supporting the robustness of this classification in diverse clinical settings (Table 3).

## Discussion

Rectal lesions larger than 3 cm are considered potentially malignant, with a high risk of underlying adenocarcinoma. Previous studies from our group demonstrated an incidence of invasive adenocarcinoma close to 20% in lesions that were preoperatively diagnosed as adenomas based on endoscopic biopsies [10, 11].

In cases of local excision of rectal lesions with submucosal invasion by adenocarcinoma (pT1), the Japanese Society for Cancer of the Colon and Rectum (JSCCR, 2019) [3] guidelines state that such resections are not considered curative when specific histopathological risk factors are identified due to the high risk of local recurrence or lymph node metastases. These adverse factors include: submucosal invasion ≥ 1000 µm, positive angiolymphatic invasion, poorly differentiated adenocarcinoma, signet-ring cell or mucinous carcinoma, tumor budding grade 2 or 3 at the deepest invasion point, or involved deep margins after endoscopic removal. Similarly, the European Society of Gastrointestinal Endoscopy (ESGE) [4] guidelines recommend considering these same factors when assessing the need for additional surgery in Western settings. Decision-making in pT1 rectal adenocarcinoma must be cautious, as large surgical series with long-term follow-up report local recurrence rates up to 22.7% (24 of 150 patients) [6].

When analyzing high-risk pathological criteria for lymph node invasion in rectal cancer, lymphovascular invasion, poor differentiation, and high-grade tumor budding are observed in fewer than 10–15% of cases [5, 12]. In the study by Leijtens et al. [6], only 29 of 150 pT1 rectal tumors (19.2%) showed at least one of these risk factors. This raises a key question for the remaining 80% of lesions: should a watch-and-wait approach be adopted, or is more aggressive surgery required? In these patients, an adequate local excision and precise submucosal invasion assessment are essential for proper clinical management.

For endoscopic or surgical excision, fragmentation must be avoided, and both lateral and deep margins should exceed 1 mm to be considered adequate [13, 14]. However, the adequacy of margin clearance between 0.1 and 1 mm has also been questioned [15].

A key prognostic factor in T1 rectal adenocarcinoma is the assessment of submucosal invasion. In pedunculated lesions, the Haggitt classification [8] defines four invasion levels, with levels I–III associated with negligible risk of nodal metastasis. Thus, these are considered low-risk. However, polypoid lesions account for only 21.3% of T1 adenocarcinomas [5]. This percentage is even lower in histological analysis, as many of these polypoid lesions lack a clear stalk, making it difficult to determine Haggitt level II [8], as illustrated in Fig. 1B.

In non-polypoid lesions, the anatomical reference used to assess submucosal invasion is the muscularis mucosae. In this context, the Kikuchi classification [16] is one of the most commonly employed systems, dividing the submucosa into three thirds: sm1, sm2, and sm3. According to this system, the risk of lymph node involvement increases significantly with depth of invasion—ranging from approximately 0–3% in sm1 to as high as 28% in sm3. However, a major limitation of this classification lies in its poor anatomical precision: while sm1 is defined as invasion up to 300 µm, sm3 is loosely described as “close” to the muscularis propria without a specific quantitative threshold, and sm2 is ambiguously situated between sm1 and sm3.

To address these anatomical ambiguities, Kudo [17] proposed dividing the submucosa into three equal layers, regardless of lesion type. Yet, this still depends on identifying the muscularis mucosae—present in only 35–45% of specimens [5, 12], as also seen in our study. Furthermore, we observed inconsistent identification among pathologists, compromising diagnostic reproducibility. To mitigate this, Ueno et al. [18] proposed a hypothetical line to estimate its position. However, this introduces further uncertainty in measurement and classification.

To reduce subjectivity in the assessment of tumor invasion, a methodology based on quantitative measurement of adenocarcinoma infiltration into the submucosa was introduced. Kitajima et al. [12] proposed a cut-off point of 1000 µm as a safety threshold after observing no lymph node metastases in cases with submucosal invasion below this depth. This criterion was subsequently adopted by both the JSCCR and ESGE as a parameter for defining curative resection. Ueno et al. [18], on the other hand, proposed a more conservative approach, setting the threshold at 2000 µm to increase sensitivity in detecting nodal risk. However, these thresholds have been the subject of ongoing debate. A recent systematic review questioned the validity of the ≥ 1000 µm cut-off as an independent risk predictor, although the authors acknowledged important methodological limitations, including heterogeneity in histopathological measurement techniques and the low quality of several of the included studies [19].

Beyond the absolute value of invasion, the main issue lies in the assumption that submucosal thickness is uniform across patients, which does not reflect anatomical reality. In fact, Kitajima et al. [12] reported significant variability in submucosal thickness at the tumor site, with a mean of 2743.3 ± 2468.5 µm and a range extending up to 23,000 µm. Similarly, Casalots et al. [5] described a mean thickness of 7130 ± 3380 µm, and in the present study, we observed a range between 1900 and 15,300 µm. This anatomical heterogeneity challenges the reliability of using fixed linear measurements as a universal prognostic criterion. A 1000 µm invasion may correspond to a superficial tumor in a thick submucosa or a deeply invasive lesion in a thinner one.

**Fig. 3:**
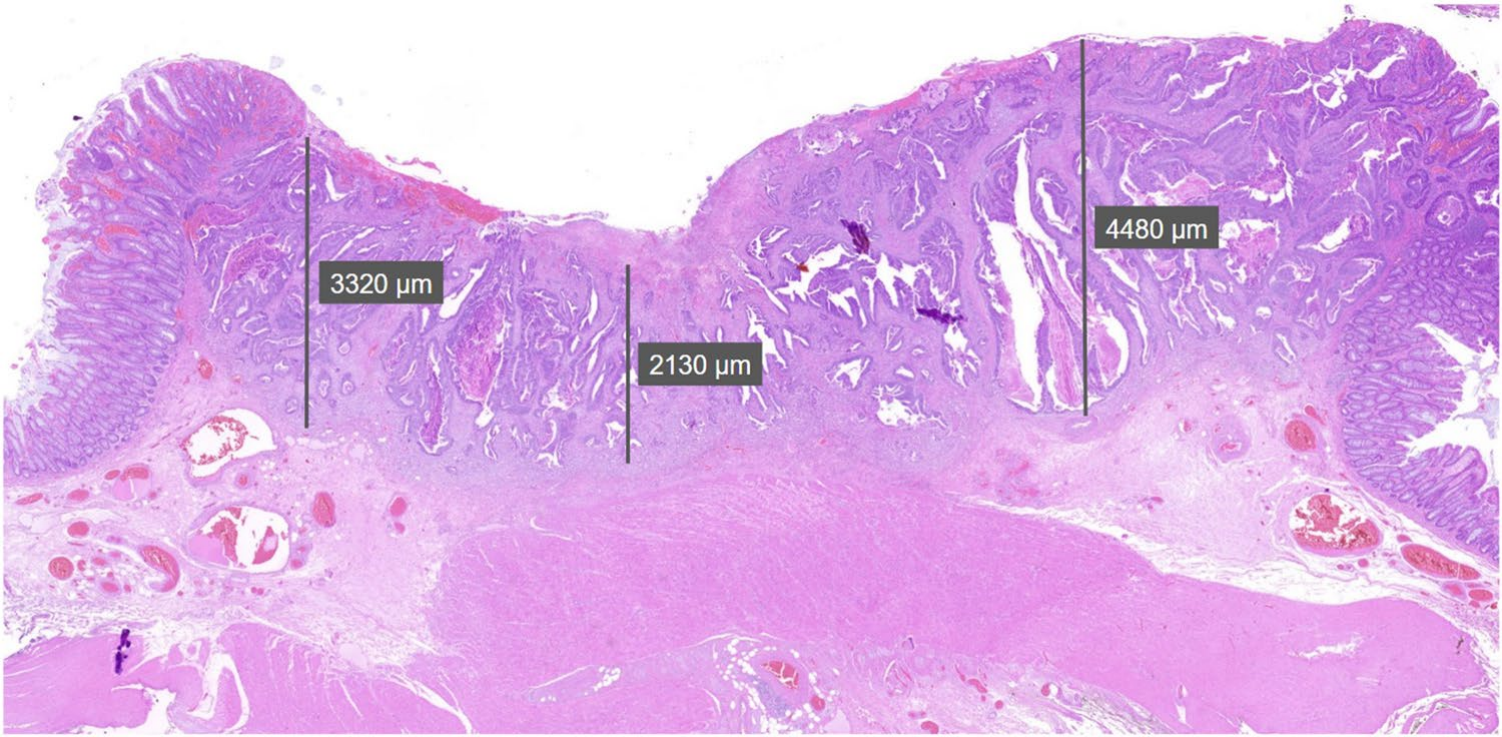
Variability in measurements without muscularis propria reference. In the absence of a consistent anatomical landmark, different surface starting points within the same lesion yield markedly variable measurements (2130–4480 µm), illustrating the risk of inconsistent invasion depth assessment

Another relevant issue is the imprecision in determining the surface reference point from which invasion depth is measured. In the absence of a consistent anatomical landmark, such as the muscularis mucosae, the same observer may select different starting points at different times. This variability can result in substantial differences in invasion depth measurements [5]. As illustrated in Fig. 3, measurements from the same histological specimen can vary widely—from 2130 to 4480 µm—effectively doubling, depending on the chosen reference point.

This lack of consistency may compromise diagnostic accuracy. Therefore, making therapeutic decisions based solely on absolute measurements—without consistent anatomical landmarks and without accounting for the relative proportion of invasion in relation to the total submucosal thickness—may lead to unnecessary overtreatment or a false sense of curative resection.

Given the limitations of traditional classifications, our group, Casalots et al. [5], proposed an alternative approach based on the consistent presence of the muscularis propria as an anatomical landmark in resected specimens. This led to the development of the Taulí-pT1 classification for rectal adenocarcinoma. This system is based on measuring tumor invasion from the muscularis propria, a structure found in virtually all full-thickness resection specimens, thereby enhancing diagnostic reproducibility. In this context, we introduced the concept of “healthy residual submucosa” (hrSB), defined as the thickness of uninvolved submucosa remaining between the tumor front and the muscularis propria. This model eliminates the dependence on polyp morphology and the presence of the muscularis mucosae, allowing for a more objective and universally applicable assessment of invasion depth. Furthermore, comparing the hrSB to the total submucosal thickness within the tumor area provides a relative value that may better reflect the biological behavior of the tumor than absolute linear measurements alone.

The primary aim of the present study was to validate the Taulí-pT1 classification as a risk stratification tool for pT1 rectal adenocarcinoma. The results demonstrated excellent interobserver agreement in measurements of total submucosal thickness, hrSB, and tumor invasion depth. These findings support the reliability and reproducibility of the Taulí-pT1 classification.

In contrast, identification of the muscularis mucosae showed moderate-to-low agreement, confirming its limited diagnostic utility in local resection specimens—not only due to its low presence, but also because of difficulties in histological identification. The subcategorization of submucosal invasion into sm1, sm2, and sm3 achieved good overall agreement among evaluators. Variations in the identification of the muscularis mucosae did not meaningfully affect the final assessments of invasion or total submucosal thickness since discrepancies were confined to seven cases, many of which did not involve substantial differences in measurement. The homogeneity in the identification of the muscularis mucosae could be enhanced by the use of specific immunohistochemical stains for muscle, such as desmin.

Regarding the analysis by professional profile, the gastrointestinal pathology specialists (Pat2-AB and Pat3-SM) demonstrated a high level of consistency with each other. The senior generalist pathologist (Pat4-JC) largely agreed with the specialists, with only minor discrepancies in borderline cases. Notably, the trainee pathologist (Pat1-CR) achieved comparable results, reinforcing the clarity and applicability of the system even in less experienced hands.

This study has several limitations. First, the analysis was confined to assessing the reproducibility of the Taulí-pT1 classification between different observers and did not evaluate its prognostic value. Future studies will be required to demonstrate its clinical applicability by correlating the proposed staging system with lymph node metastasis and local recurrence, as well as other clinical and survival parameters.

Second, although the sample size of 30 is within the acceptable range for interobserver agreement studies, its single-center design and limited number of cases inevitably restrict generalizability [9]. The number of evaluators was limited to four pathologists. While the selection intentionally included a representative range of experience levels, a larger pool of observers could have provided a broader perspective on the reproducibility of the system across different contexts. Larger, multicenter or even international studies are required to validate our findings in broader and more diverse clinical settings. This need for external validation has also been highlighted in previous inter-observer studies of pT1 colorectal cancer substaging [20], which emphasized that reproducibility and applicability of new histological approaches must be confirmed across institutions.

Third, although we initially considered performing parallel measurements using other classification systems (such as Kikuchi classification or absolute depth of invasion), we ultimately limited our analysis to the Taulí-pT1 parameters. The aim of the study was not to demonstrate superiority, but rather to validate the interobserver reproducibility and internal consistency of the proposed method. Future studies with larger cohorts should include head-to-head comparisons with established systems in order to determine its relative prognostic value.

Fourth, with the data obtained in this study, which was limited to reproducibility analysis in a small single-center cohort, we did not have sufficient statistical power to evaluate how hrSB measurement could be integrated with other known risk factors such as lymphovascular invasion or tumor budding. Indeed, in our series, these features were present in fewer than 20% of cases (Table 1), which prevented meaningful analysis. The main rationale for standardizing this classification is to improve therapeutic decision-making in the majority of patients, where these adverse features are absent. The development of composite predictive models that integrate hrSB measurement with other pathological parameters will therefore require future studies specifically designed and powered to address their combined prognostic impact.

Fifth, because the method requires specimens containing the muscularis propria, its use is currently limited to full-thickness resection specimens. This trade-off between improved histological assessment and the increased invasiveness of full-thickness resections highlights the need to carefully balance diagnostic accuracy against procedural morbidity when defining management strategies. Nevertheless, if future studies confirm the prognostic significance of this parameter, it would be worth considering the risk–benefit balance of standardizing full-thickness resections in selected cases to provide more accurate histopathological information to guide clinical management.

In addition, the study relied on a preselected histological section with the smallest residual healthy submucosa thickness, and all measurements were performed on this digitized image. In routine practice, different pathologists may not always select the same section with the minimum hrSB, although the increasingly widespread use of digital pathology, which facilitates precise measurements, helps to mitigate this limitation. Building on this, the relatively standardized nature of hrSB measurement suggests that the Taulí-pT1 classification could benefit in the future from integration with AI-assisted histological analysis. Automated image processing could help identify the point of closest tumor proximity to the muscularis propria and calculate the proportion of submucosal invasion, potentially increasing consistency and reproducibility across observers with different levels of experience. Although still exploratory, this represents a promising avenue for future research to further improve standardization and clinical applicability.

Overall, these limitations highlight the need for larger, multicenter studies with comprehensive pathological and clinical data to fully establish the prognostic utility of the Taulí-pT1 classification.

## Conclusions

The Taulí-pT1 classification demonstrated excellent interobserver reproducibility in the measurement of healthy residual submucosa, total submucosal thickness, and tumor invasion, even among pathologists with varying levels of experience. These findings support its applicability as an objective and standardized tool for risk stratification in pT1 rectal adenocarcinomas.

## Supplementary Information

Below is the link to the electronic supplementary material.

## Supplementary Information

Below is the link to the electronic supplementary material.

Supplementary file 1 (MP4 195 MB)

## Data Availability

The full dataset supporting the findings of this study is provided as a supplementary Excel file submitted alongside the manuscript. Further information is available from the corresponding author upon reasonable request.

## Declarations

### Permission to reproduce material from other sources

No permission was required to reproduce material from other sources.
